# Histone deacetylase inhibitors induce invasion of human melanoma cells in vitro via differential regulation of N-cadherin expression and RhoA activity

**DOI:** 10.1186/s12885-016-2693-3

**Published:** 2016-08-22

**Authors:** María Díaz-Núñez, Alejandro Díez-Torre, Olivier De Wever, Ricardo Andrade, Jon Arluzea, Margarita Silió, Juan Aréchaga

**Affiliations:** 1 Laboratory of Stem Cells, Development & Cancer, Department of Cell Biology & Histology, Faculty of Medicine & Nursing, University of the Basque Country (UPV/EHU), Leioa, Biscay Spain; 2 Analytical & High Resolution Biomedical Microscopy Core Facility, University of the Basque Country (UPV/EHU), Leioa, Spain; 3 Laboratory of Experimental Cancer Research, Department of Radiotherapy and Experimental Cancer Research, Ghent University Hospital, Ghent, Belgium; 4 Department of Cell Biology & Histology, Faculty of Medicine & Dentistry, University of the Basque Country, E-48940 Leioa, Spain

**Keywords:** Histone deacetylase inhibitors, HDACi, Melanoma, Cell invasion, N-cadherin, RhoA

## Abstract

**Background:**

Histone deacetylase inhibitors (HDACi) exert multiple cytotoxic actions on cancer cells. Currently, different synthetic HDACi are in clinical use or clinical trials; nevertheless, since both pro-invasive and anti-invasive activities have been described, there is some controversy about the effect of HDACi on melanoma cells.

**Methods:**

*Matrigel* and Collagen invasion assays were performed to evaluate the effect of several HDACi (Butyrate, Trichostatin A, Valproic acid and Vorinostat) on two human melanoma cell line invasion (A375 and HT-144). The expression of N- and E-Cadherin and the activity of the RhoA GTPase were analyzed to elucidate the mechanisms involved in the HDACi activity.

**Results:**

HDACi showed a pro-invasive effect on melanoma cells in vitro. This effect was accompanied by an up-regulation of N-cadherin expression and an inhibition of RhoA activity. Moreover, the down-regulation of N-cadherin through blocking antibodies or siRNA abrogated the pro-invasive effect of the HDACi and, additionally, the inhibition of the Rho/ROCK pathway led to an increase of melanoma cell invasion similar to that observed with the HDACi treatments.

**Conclusion:**

These results suggest a role of N-cadherin and RhoA in HDACi induced invasion and call into question the suitability of some HDACi as antitumor agents for melanoma patients.

## Background

Gene expression in eukaryotic cells is epigenetically regulated by the antagonistic activities of histone acetyltransferases (HATs) and histone deacetylases (HDACs). Histone acetylation by HATs mediates the formation of accessible chromatin regions and promotes gene expression, whereas histone deacetylation catalyzed by HDACs leads to a repressive state due to chromatin compaction. Given the capital function of these enzymes, their activities are tightly regulated.

The role of HDACs in cancer progression was first demonstrated in acute promyelocytic leukemia [1] and is currently known in many other tumor types, including melanoma [2–5]. Therefore, HDACs have emerged as a target for anticancer therapies and many authors have focused their research on this issue. As a consequence, it has been demonstrated that several HDAC inhibitors (HDACi) induce growth arrest, differentiation and apoptosis in different cancer cell lines [6–8]. Several HDACi have been previously used in clinical trials with different tumor types, including melanoma [9]. In a phase I/II clinical trial of Valproic acid in combination with Topoisomerase I inhibitor, Karenitecin, 47 % of patients had stable disease with median progression-free survival of 10.3 weeks versus 34 % with stable disease with median progression-free survival of 7.9 weeks shown in patients on a previous phase II study with Karenitecin as a single agent [10]. In another phase I study with the HDACi MS-275 disease stabilization and partial remission were observed in patients with melanoma and other solid tumors [11]. Phase I clinical trials of vorinostat in combination with other drugs have shown interesting results on melanoma patients, with a significant percentage of disease stabilization and tumor measurement reduction. Nevertheless, no responses were shown under RECIST criteria [12].

HDACi have also been associated with the epithelial-to-mesenchymal transition (EMT) [13, 14], a process that contributes to invasion and progression of cancer cells. This process is characterized by the acquisition of an elongated fibroblast-like morphology, inhibition of cell adhesion and an increase of cell motility as one of its main hallmarks [15, 16].

HDACi can be classified into several structural classes including, in order of decreasing potency, hydroxamic acids, cyclic peptides, benzamides and aliphatic acids. Sodium butyrate is a fatty acid synthesized by intestinal bacteria that inhibits the cell cycle through the activation of the p21Waf1/Clip1 gene [17]. We have previously shown that butyrate induces the apoptosis of melanoma cells in a synergistic manner when combined with resveratrol [6]. In spite of the clear pro-apoptotic effect of butyrate and other HDACi, its effect on the invasive capability of cancer cells is still controversial. Some authors point to butyrate as an inhibitor of invasion in cancer cells [18–20], whereas others have observed the opposite result [16, 21]. Therefore, in order to characterize the effect of HDACi on human melanoma cells, we have evaluated the invasiveness of two human melanoma cell lines (A375, derived from a primary tumor, and HT-144, obtained from a subcutaneous metastatic site) treated with a variety of HDACi, two of them were hydroxamic acids (Trichostatin A and Vorinostat) and the other two were aliphatic acids (Butyrate and Valproic acid). We have also analyzed the effect of these inhibitors on cadherin expression and RhoA activity. Our results demonstrate that most HDACi promote melanoma cell invasion in vitro after 24 h. Moreover, this pro-invasive response to HDACi could be mediated by the E- to N-cadherin switch at the cell-cell adhesion complexes and a decrease in the activity of RhoA small GTPase.

## Methods

### Cell culture

A375 and HT-144 melanoma cell lines were purchased from ATCC. Cells were grown in DMEM growth medium supplemented with 10 % Fetal Bovine Serum, 1 % L-Glutamine and 1 % Penicillin-Streptomycin. Cells were maintained at 37 °C in an incubator with a 5 % CO_2_ humidified atmosphere, and were subcultured every 2–3 days with trypsin-EDTA solution.

### Drug treatments

Melanoma cells were treated with 2 mM butyrate (Sigma-Aldrich, St. Louis, MO), 100 nM TSA (Sigma-Aldrich, St. Louis, MO), 1 mM valproic acid (Sigma-Aldrich, St. Louis, MO) and 3 μM vorinostat (Selleckchem, Houston, TX). In order to minimize the interference of apoptosis in the quantification of cell invasiveness we have used low concentrations of HDACi among the standard range found in the literature [6, 22–29]. The cells were exposed to the HDACi during 24 h in all the experiments except if something different is specified.

### Drug sensitivity assay

**Fig. 1:**
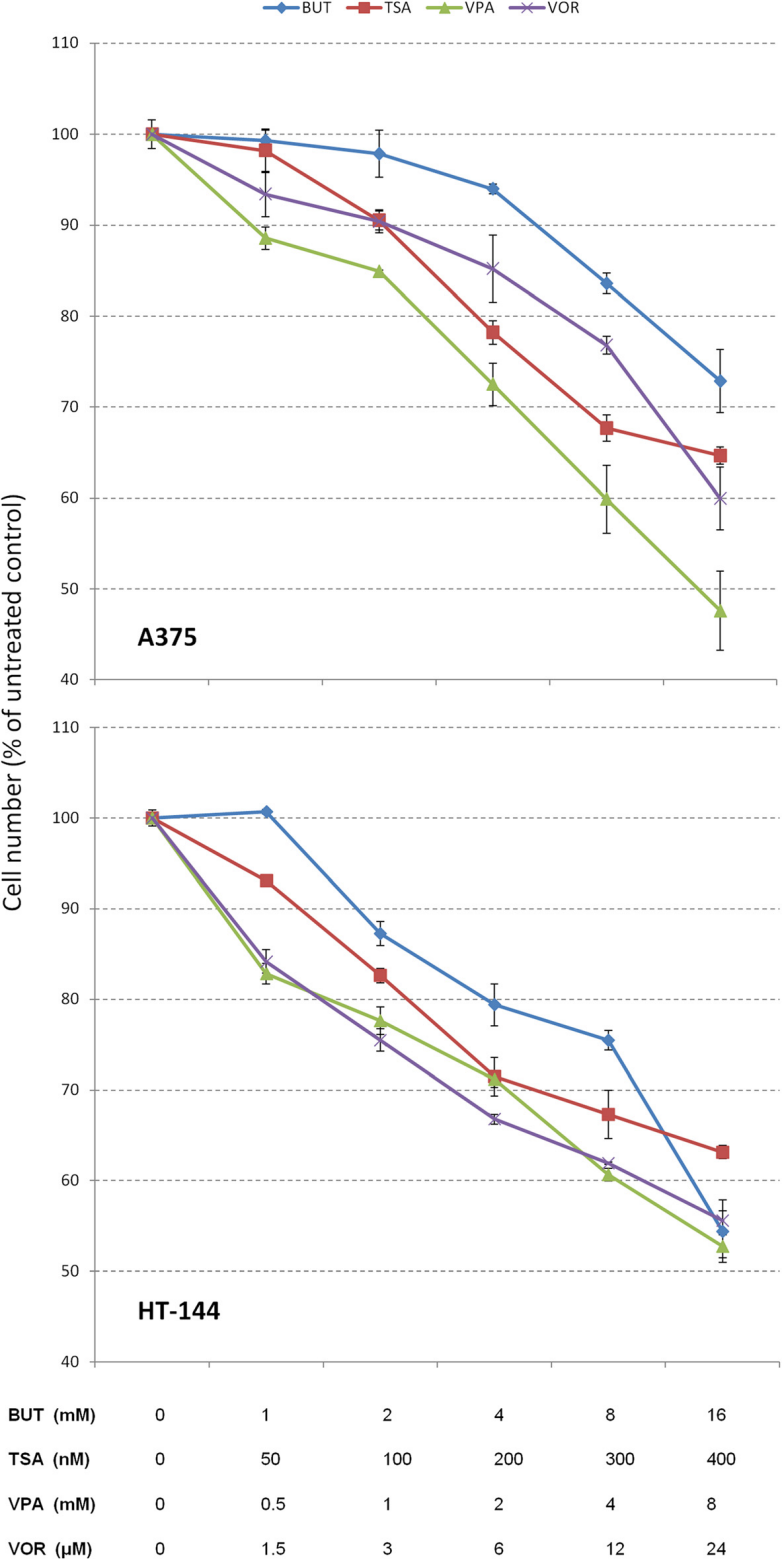
HDAC inhibitor sensitivity assay. The number of viable A375 cells (*upper panel*) was reduced by Butyrate 2 mM (by 3 %), TSA 100 nM (by 10 %), Valproic acid 1 mM (by 15 %) and Vorinostat 3 μM (by 10 %) compared with the untreated control (set at 100 %). HT-144 cells (*lower panel*) were more sensitive to HDACi and its viability was reduced by Butyrate 2 mM (by 13 %), TSA 100 nM (by 17 %), Valproic acid 1 mM (by 22 %) and Vorinostat 3 μM (by 25 %). Cell number was determined after 24 h of treatment by the MTT proliferation assay (Sigma). Shown data correspond to representative experiments carried out in triplicate and repeated twice. Values are expressed as the mean ± SD

A375 and HT144 cells were seeded at 10,000 cells/well and 5000 cells/well respectively in triplicate in 96-well plates and treated for 24 h with varying concentrations of Butyrate, TSA, Valproic acid and Vorinostat (Fig. 1). After 24 h, relative viable cell numbers were determined using the MTT viability Assay (Sigma), which measures bioreduction of MTT into a soluble formazan that was measured in a microplate reader at A540 nm.

### Flow cytometric analysis of apoptosis

A375 melanoma cells were treated with HDAC inhibitors as indicated above during 24 and 48 h, cells were then harvested, washed and stained using the apoptosis detection kit Annexin V FITC of Immunostep (Salamanca, Spain) according to the manufacturer’s recommendations. Samples were acquired on a Gallios flow cytometer (Beckman Coulter, Brea, CA). Acquired samples were analyzed using Kaluza software (Beckman Coulter, Brea, CA).

### *Matrigel* invasion assay

*Matrigel* is an extracellular matrix component similar to the basal membrane that separates epidermis and dermis so we decided to use this assay to evaluate the invasiveness of a primary tumor derived cells (A375). We used 6.5 mm diameter Transwell inserts (Costar) with 8 μm pore membranes. The membranes were coated with 35 μl Matrigel (BD Biosciences) at 3 mg/ml in serum-free DMEM and allowed to solidify in the incubator at 37 °C for 2 h. Cells were detached, washed twice with PBS and re-suspended in serum-free DMEM. 5×10^5^ cells in 50 μl were placed in the upper chamber with the corresponding treatment, and the lower chamber was filled with 1 ml of DMEM-10 % FBS. After a 24 h incubation period, the cells that remained in the upper chamber were scraped away. Cells in the lower surface of the membrane were stained with Hoechst for 15 min. Pictures of the lower surface of the insert were taken with a confocal microscope (Olympus Fluoview FV500) using a 4× objective capturing the central area of the membrane (9 mm^2^). Invading cell number was quantified with the *ImageJ* software.

### Collagen invasion assay

Type I collagen is the most abundant component of the connective tissue of the dermis so it was used to analyze the invasion of cells derived from a subcutaneous metastatic site (HT-144). The type I collagen solution was prepared mixing the following components at 4 °C: four volumes of type I collagen (3.49 mg/ml), five volumes of calcium-magnesium-free Hank’s balanced salt solution, one volume of MEM (10×), one volume of 0.25 M NaHCO3, 2.65 volumes of culture medium, and 0.3 volumes of 1 M NaOH. 1.25 ml of type I collagen solution was added to each well of six-well plates, homogeneously spread, and solidified for one hour at 37 °C on a flat surface in a humidified atmosphere with 5 % CO_2_. 10^5^ single cells suspended in 1 ml of culture medium with the corresponding treatment were seeded on top of the type I collagen gel and maintained at 37 °C in an incubator.

Cell morphology was studied and invasion was scored after 24 h of incubation. The number of invasive and noninvasive cells was counted in ten randomly selected microscopic fields with a 20× objective using an inverted phase contrast microscope (Nikon Eclipse Ti-S). The invasion index was calculated as the ratio of the number of invading cells, which showed dark protrusions in their membrane, divided by the number of non-invasive cells counted in each field. Then, control was set as 100 and the other data relative to control. For the phalloidin staining collagen gels were fixed with 3 % paraformaldehyde, permeabilized with 0.5 % Triton, and then incubated with Phalloidin-TRITC and DAPI for 30 min. Actin cytoskeleton images were taken with a confocal microscope (Olympus Fluoview FV500).

### Shape factor

Pictures of phalloidin stained HT-144 cells invading collagen after 24 h of culture (with or without HDACi) were taken with a confocal microscope (Olympus Fluoview FV500) at low magnification (10× objective). Then shape factor, or circularity factor, was measured with Image J as 4 πA/P2, with A being the area and P the perimeter of the cell. Shape factor is measured from 0 to 1. A shape factor of 1 corresponds to a round cell, as shape factor goes to zero cells are assumed to be increasingly more spread. Ten pictures of three independent experiments were evaluated for each condition.

### Protein extraction and Western Blot

Cells were lysed in 1× Laemmli buffer and protein concentrations were determined via Bio-Rad Rc-Dc protein assay in accordance with the manufacturer’s instructions. Twenty-five nanogram of proteins were transferred to PVDF membranes. The membranes were probed with the corresponding antibodies, incubated with diluted HRP-linked secondary antibody and visualized by enhanced chemiluminescence (ECL) in accordance with the recommended procedure.

### Immunoprecipitation

To study the function of cadherins we performed immunoprecipitation with α-catenin in order to precipitate proteins in the cadherin/catenin complex from cell extracts of untreated and HDACi treated (24 h) cultures. Immunoprecipitation for α-catenin was carried out with magnetic beads (Dynabeads M-280 Sheep anti- Rabbit IgG). Magnetic beads were incubated with α-catenin antibody for 2–4 h at 4 °C. After antibody incubation, cell lysates, made in NP40 lysis buffer (1 % NP40 PBSD+), were added and incubated overnight at 4 °C. Then beads were washed five times for 5 min and finally denatured in boiling Laemmli buffer (1 ml Laemmli 1.5×, 75 μl β-mercaptoethanol y 75 μl bromophenol blue). Finally, cadherins and catenins were detected by Western blot.

### Rho pull down assay

GTP-bound Rho GTPase was pulled down with rhotekin RBD protein GST beads (Cytoskeleton Inc, Denver, CO) according to the manufacturer’s instructions from cell lysates of A375 cells untreated or treated for 48 h with HDACi. Cells were lysed in PIPES 50 mM at pH 7 (0.130 mM NaCl, 1 mM PMSF, 1 mM DTT, 5 μl/ml leupeptin and 0,5 % Triton X-100). Later, samples were diluted to 0.5 mg/ml in lysis buffer (50 mM Tris pH 7.5, 10 mM MgCl_2_, 0.3 M NaCl, 2 % IGEPAL) with a protease inhibitor cocktail. Samples were centrifuged at 14,000 rpm at 4 °C for 15 min and 1/10th volume of loading buffer was added (150 mM EDTA). GTPγS 0.2 mM and GDP 1 mM were added to two control lysates as positive and negative control respectively.

Samples were incubated 15 min at room temperature and reaction was stopped with stop buffer (600 mM MgCl_2_) to a final concentration of 60 mM. Then, protein beads were resuspended, 20 μl were added to each tube, and gently rotated at 4 °C for 1 h. Beads were washed twice with 500 μl of wash buffer (25 mM Tris pH 7.5, 30 mM MgCl_2_, 40 mM NaCl) and centrifuged at 5000 rpm for 1 min a 4 °C. Finally, beads were resuspended in SDS buffer and analyzed by Western Blot using a RhoA specific monoclonal antibody.

### Expression constructs

HT-144 cells were cotransfected with two vectors encoding RhoA dominant negative (RhoA-T19N) and the green fluorescent protein (GFP) at a 5:1 ratio. 48 h after transfection, the percentage of GFP expressing cells was analyzed by flow cytometry to evaluate transfection efficiency. Transfection was performed using Xfect (Clontech) following the manufacturer’s instructions. The collagen invasion assay was performed 48 h after transfection.

### Antibodies

Antibodies against E-cadherin (HECD1) and RhoA were acquired from Takara and Upstate respectively. Antibodies for α-catenin and N-cadherin were purchased from Sigma. The neutralizing antibody against N-cadherin GC-4 was also acquired from Sigma.

### Gene silencing

2,5×10^5^ HT-144 cells were plated in 6-well plates and 24 h later transfected with iRNAmax Lipofectamine and siRNAs targeting N-cadherin [30], purchased to Qiagen: Inhibition of N-cadherin expression was achieved by RNA interference using a 1:1 mixture of the following double-stranded oligoribonucleotides (1,5 μl each from 10 μM solution in 9 μl of Lipofectamine): siN-CAD2 5-AGUGGCAAGUGGCAGUAAA-3 and siN-CAD3 5-GGAGUCAGCAGAAGUUGAA-3’. To verify the specificity of the silencing effect we also used a sequence with no known mammalian target as a control (con 5-UUCUCCGAACGUGUCACGU-3). Forty-eight hours after transfection collagen invasion assays were performed in the same conditions as those used with no transfected cells. Cells lysates were obtained to confirm silencing.

### Statistical analysis

Values in the figures are expressed as means ± S.D. Statistical analyses were conducted using the Student t-test for the comparison of two data groups with GraphPad (Prism4).

## Results

### HDACi sensitivity assay

Cells were treated for 24 h with varying concentrations of Butyrate (1, 2, 4, 8 and 16 mM), TSA (50, 100, 200, 300 and 400 nM), Valproic acid (0.5, 1, 2, 4 and 8 mM) and Vorinostat (1.5, 3, 6, 12 and 24 μM). Viable cell numbers were determined by colorimetric assay, and plotted relative to untreated control cells (Fig. 1). At the concentrations selected for the invasion assays HDACi reduced the number of viable cells in both A375 (by 3–15 %) and HT-144 (by 13–25 %) melanoma cells. HT-144 cells were more sensitive to all HDACi than A375 cells (Fig. 1).

### HDACi induce melanoma invasion in Matrigel and type I collagen

**Fig. 2:**
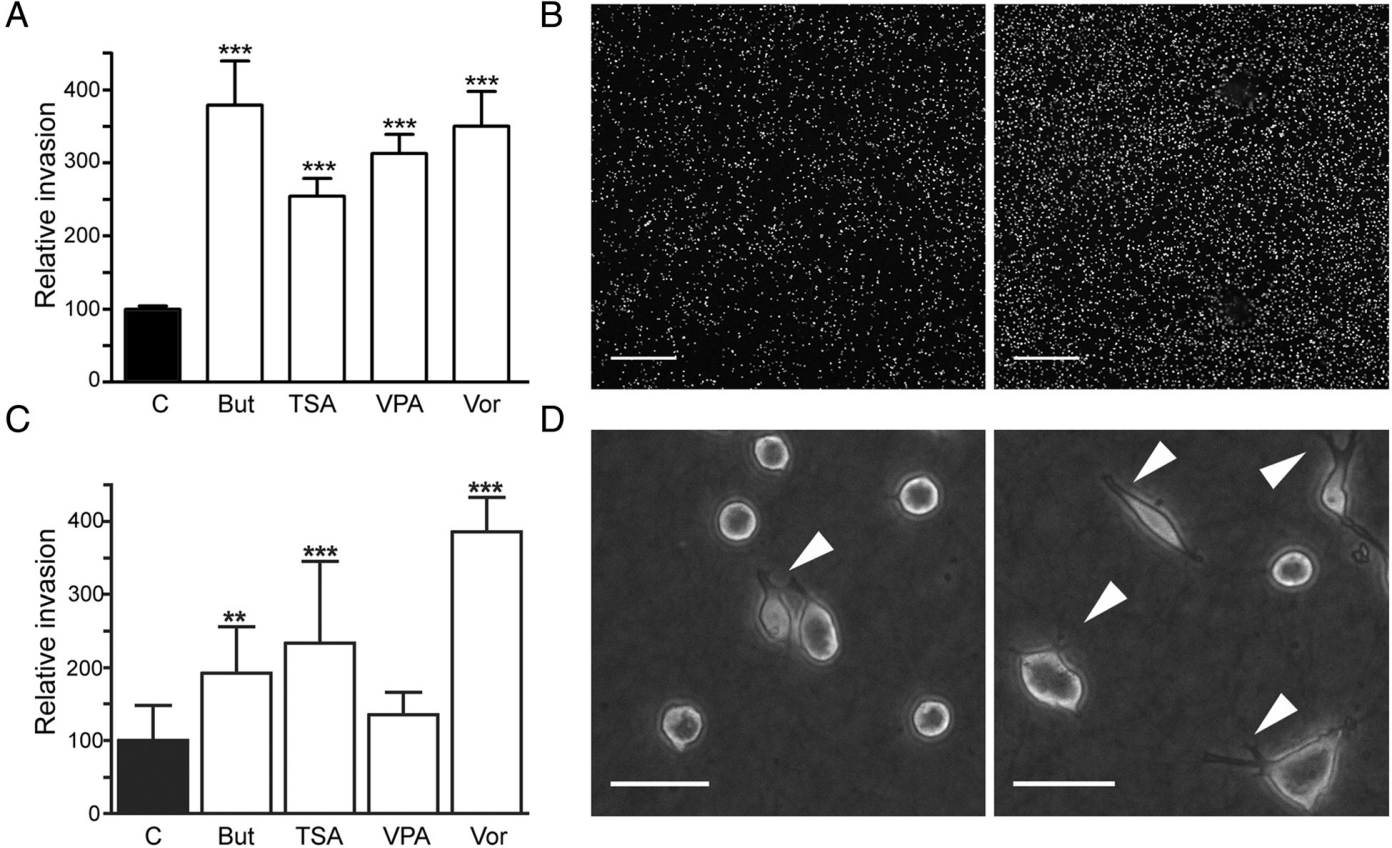
Invasion assays. **a** *Matrigel* invasion assay with the A375 cell line. Cells were treated with butyrate, TSA, Valproic acid and Vorinostat during 24 h. All HDACi significantly increased melanoma cell invasion. **b** Confocal microscopy of the transwell membrane with A375 cells stained with Hoechst, corresponding to control (*left*) and butyrate treatment (*right*) from *Matrigel* invasion assay. Scale bar, 500 μm. **c** Type I collagen invasion assay with HT-144 cell line. Cells were treated with the same agents as in (**a**) for 24 h. Butyrate, TSA and Vorinostat induced a significant increase on invasion, whereas Valproic acid had no effect. **d** Contrast phase microscopy of HT-144 cells on collagen gel, corresponding to control *(left*) and vorinostat (*right*) after 24 h treatment. Scale bar, 50 μm. Statistically significant differences from control; ***p < 0.001, ** *p* < 0.01 and * *p* < 0.05. Abbreviations: But, butyrate; TSA, Trichostatin A; VPA, Valproic acid; Vor, Vorinostat

In order to elucidate the role of HDACi in the invasive ability of melanoma cells, we have tested the effect of four different HDACi on A375 and HT-144 melanoma cell lines using *Matrigel* and type I collagen invasion assays. Our results show that A375 cell invasion is significantly increased in *Matrigel* when treated with butyrate, TSA, valproic acid or vorinostat during 24 h (*p* < 0.001) (Fig. 2 a). In contrast, HT-144 cells treated with butyrate (*p* < 0.01), TSA (*p* < 0.001) and vorinostat (*p* < 0.001) show significantly higher invasion into type I collagen when compared to untreated cultures (Fig. 2 c). Nevertheless, HT-144 cell invasiveness does not undergo significant changes in response to valproic acid (Fig. 2 c).

### HDACi induce apoptosis in melanoma cells

To analyze the induction of apoptosis by the HDACi at the selected concentrations we determined the number of Annexin V stained cells after 24 and 48 h of treatment. The number of cells undergoing apoptosis at 24 h in the untreated A375 culture was 11.7 %, this value was similar, or even lower, in the cultures treated with TSA (10.4 %) and valproic acid (7.4 %) but it was slightly increased with butyrate (17.2 %) and vorinostat (15.3 %). The effect of HDACi on apoptosis induction was higher after 48 h, the basal level of apoptotic cells observed in the untreated A375 cells (12.6 %) was significantly increased in the cultures treated with butyrate (18.1 %), valproic acid (20.4 %) and vorinostat (27 %). TSA (11 %) was the only inhibitor that did not show significant changes in the apoptosis levels after 48 h.

### HDACi treatment leads to changes in HT-144 melanoma cell morphotype

**Fig. 3:**
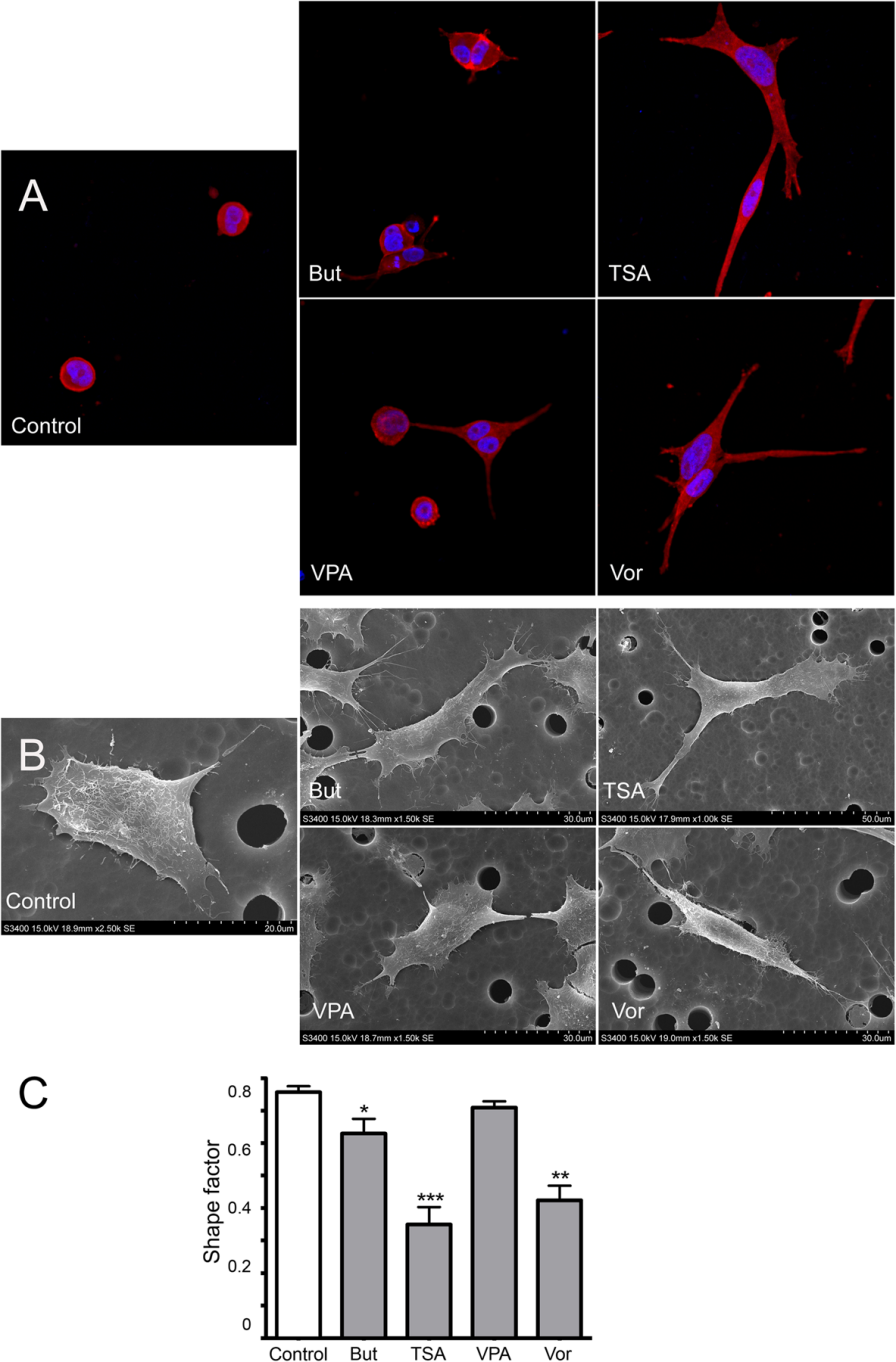
Morphological changes induced by HDACi. **a** Phalloidin stained HT-144 cells growing in collagen gel after a 24 h treatment with butyrate, TSA, Valproic acid and Vorinostat. **b** SEM images of A375 invading cells treated with the same agents. All HDACi provoked morphological changes in melanoma cells, which in particular, appeared to be more elongated. **c** Shape factor, which is around 0.8 in the control, decreases to almost 0.3 with some of the inhibitors. Cells treated with valproic acid did not show a significant decrease in shape factor compared to control. Statistically significant differences from control: ***p < 0.001, ** *p* < 0.01 and * *p* < 0.05. Abbreviations: But, butyrate; TSA, Trichostatin A; VPA, valproic acid; Vor, vorinostat

After 24 h of treatment with any of the different HDACi, HT-144 cells exhibited a more elongated shape than their untreated counterparts (Fig. 3). In untreated cultures, HT-144 cells were mostly round-shaped and just about 20 % of the cells appear slightly elongated. However, the proportion of elongated cells increased up to 75 % after exposure to HDACi. We also observed that the intensity of the effect of each inhibitor on the morphology of melanoma cells was heterogeneous. Thus, butyrate and valproic acid induced subtle cell elongation at 24 h, while TSA and vorinostat had a dramatic effect on the morphotype, with the presence of long projections and mesenchymal-like cell appearance. Shape factor, which is around 0.8 in the control, decreased to almost 0.3 in the presence of some of the inhibitors. TSA and vorinostat had the strongest effect on shape factor with average values of 0.34 and 0.42 respectively, whereas butyrate (0.62) elicited a milder response. In contrast, valproic acid was the only inhibitor that did not induce a significant effect on shape factor. It should be taken into account that the percentage of elongated cells differed among the different treatments. Thus, it was around 40 % in butyrate or TSA treated cultures whereas it reached 80 % of total cells in response to vorinostat.

### HDAC inhibitors increase N-cadherin expression in melanoma cells

**Fig. 4:**
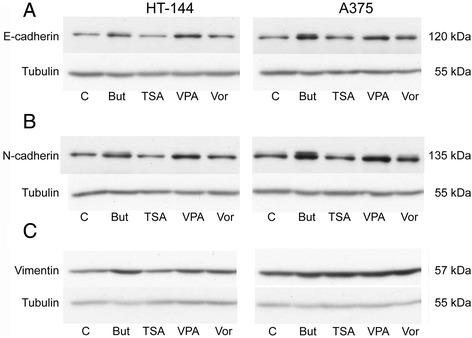
Western Blot analysis of E-cadherin and N-cadherin in HT-144 and A375 melanoma cell lines treated with HDACi during 24 h. Figure shows images from a representative experiment. Both E- and N-cadherin upregulation was observed in the two cell lines, specially with butyrate and valproic acid. Vimentin expression was induced by the four inhibitors in A375 cells but only butyrate had a significant effect on HT-144 cell line. Tubulin was used as a loading control to normalize protein content. Abbreviations: But, butyrate; TSA, Trichostatin A; VPA, valproic acid; Vor, vorinostat.

The switch from E-cadherin to the mesenchymal cell associated N-cadherin has often been observed during cancer progression. Therefore, the expression of both E- and N-cadherin was analyzed by Western blot in HT-144 and A375 cell lines treated with HDACi. The expression of the two analyzed cadherins was significantly increased in both cell lines in response to 24 h treatments with butyrate and valproic acid, with a remarkable effect on HT-144 cells (Fig. 4). In contrast, TSA and vorinostat had a very slight or null effect on cadherin expression levels.

**Fig. 5:**
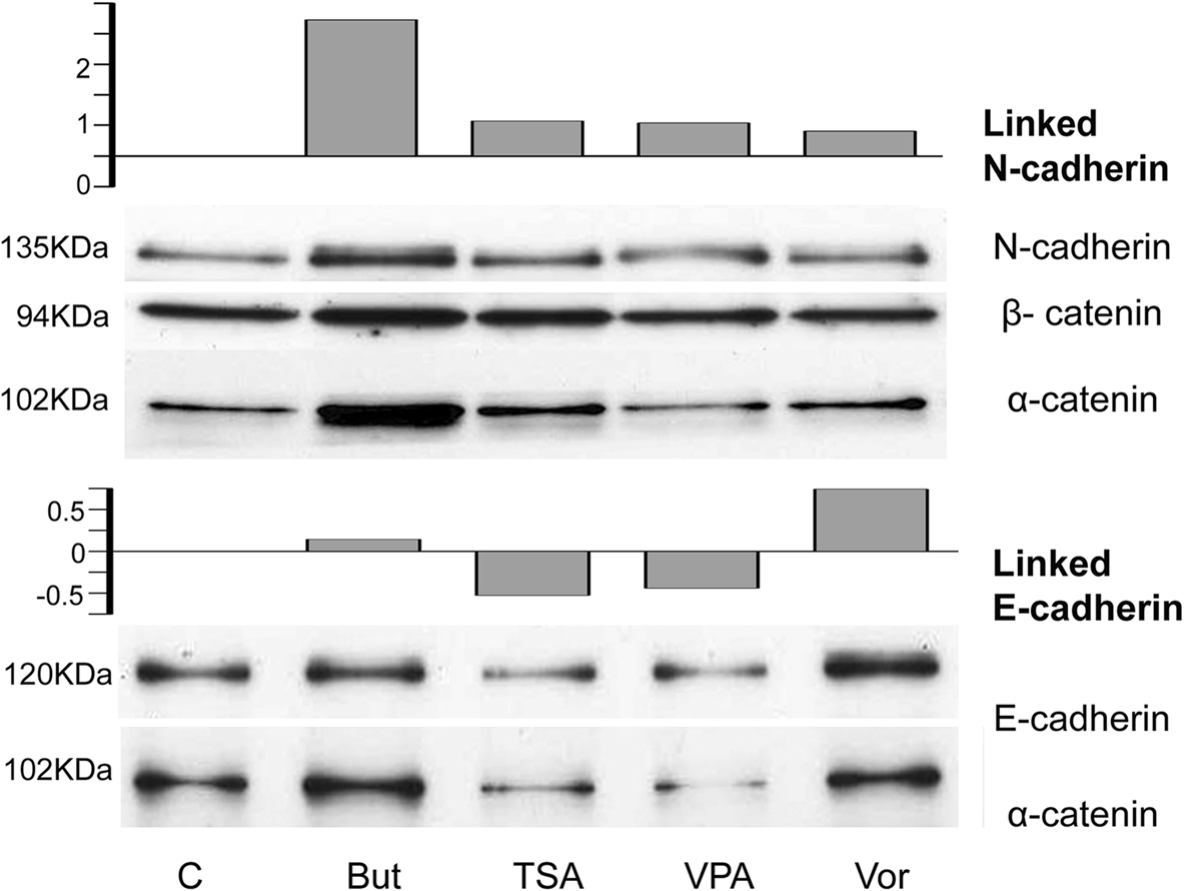
Immunoprecipitation with α-catenin. Western blot of HT-144 extracts (control and treated with HDACi for 24 h) to detect E-cadherin and N-cadherin immunoprecipitated with α-catenin. Graphs show relative intensity from E-cadherin/ α-catenin and N-caherin/ α-catenin, numeric data was obtained by densitometry (*ImageJ*). Abbreviations: But, butyrate; TSA, Trichostatin A; VPA, valproic acid; Vor, vorinostat

In order to analyze the functionality of E- and N-cadherin, we determined their association with α-catenin through immunoprecipitation (Fig. 5). Our results show that, even though the expression of total E-cadherin is up-regulated by butyrate and valproic acid, these inhibitors induce a switch from E-cadherin to N-cadherin in the association with the α-actin cytoskeleton through α-catenin. The increase of α-catenin-associated N-cadherin is especially relevant in melanoma cells treated with sodium butyrate.

### The inhibition of N-cadherin abrogates HDACi induced invasion

**Fig. 6:**
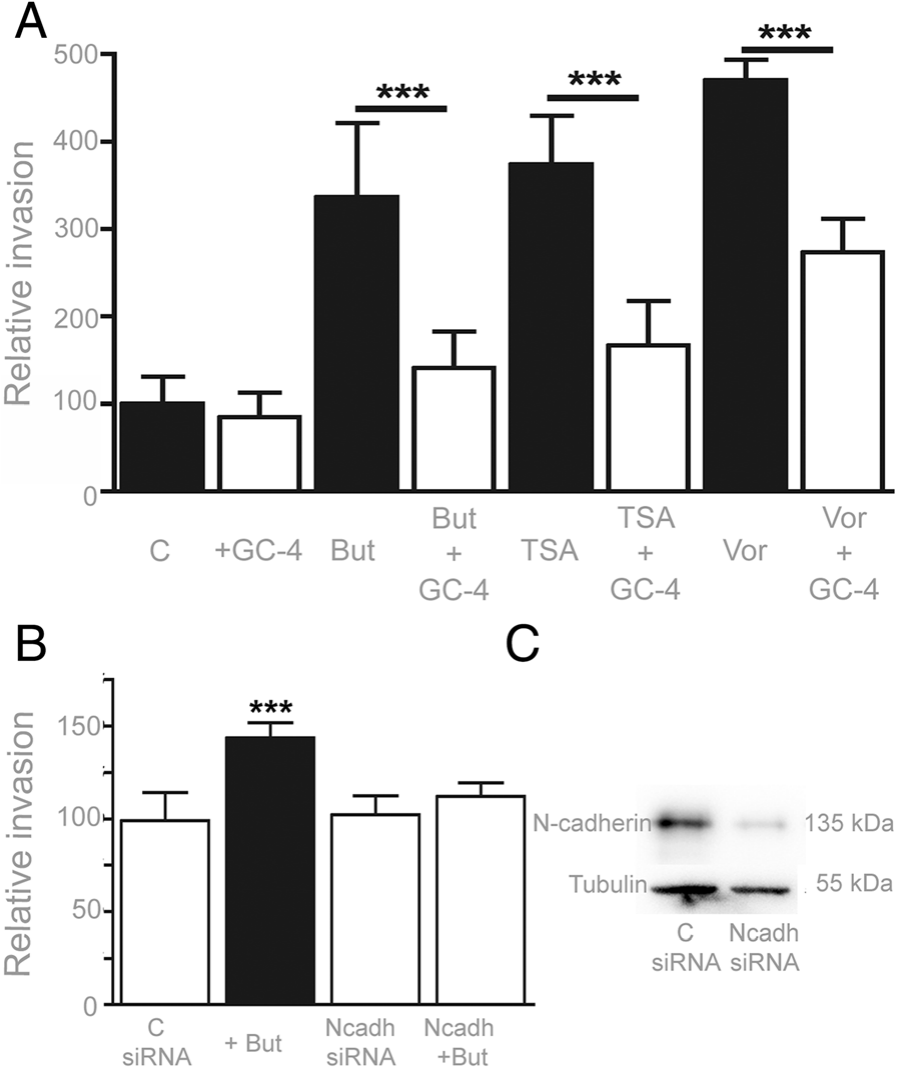
**a** Collagen invasion assay with HT-144 treated with the GC-4 antibody to block N-cadherin. When N-cadherin was blocked with GC-4 the pro-invasive effect of butyrate, TSA and Vorinostat was reverted. **b** HT-144 cells 48 h after silencing with negative control siRNA and N-cadherin (with and without butyrate) (**c**) Western Blot analysis of N-cadherin in negative control and N-cadherin siRNA transfected cells. *** Statistically significant difference from control, *p* < 0.001

To demonstrate the direct implication of N-cadherin in HDACi-induced invasion, we performed collagen invasion assays with melanoma cells treated with butyrate, TSA and vorinostat, while blocking N-cadherin with an anti-N-cadherin monoclonal antibody (GC-4 mAb) (Fig. 6 a). In these experiments, the pro-invasive effect of the HDACi was completely abrogated when the blocking antibody was added to the culture medium. Additionally, the silencing of N-cadherin using specific siRNA in HT-144 cells abrogates the butyrate-induced invasion in a similar way to that observed with blocking antibodies, which constitutes an additional evidence for N-cadherin to be a mediator of HDACi-induced invasion. Interestingly, neither the blocking antibodies nor the siRNA have any effect on melanoma cell basal invasion (Fig. 6 b). This result suggests that the basal invasion observed in untreated cells does not depend on N-cadherin and that the proinvasive effect of HDACi might be due to some signaling triggered by the disturbance of the cadherin balance in favor to N-cadherin.

### RhoA downregulation induces HT-144 cell invasion

**Fig. 7:**
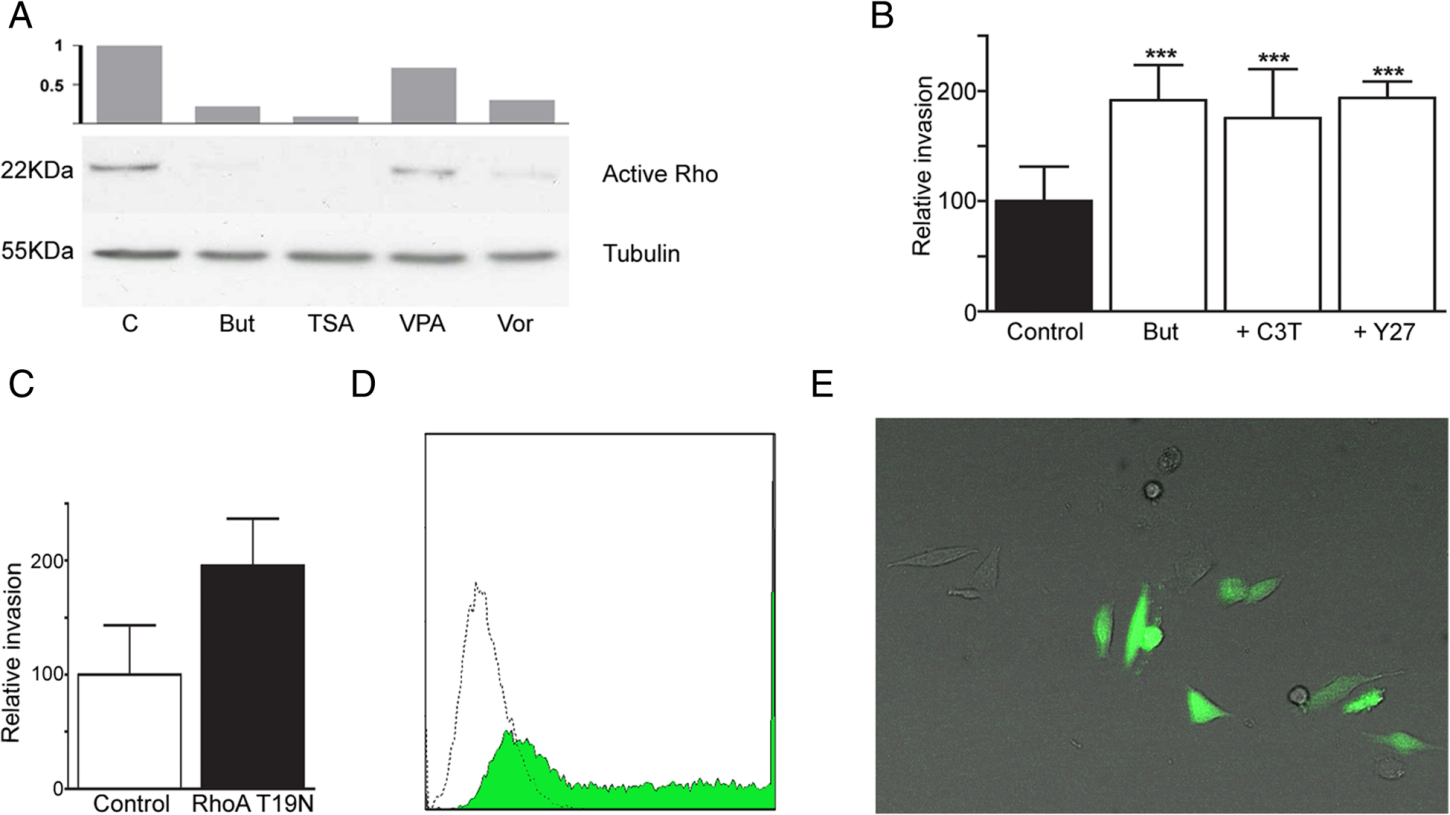
Rho implication in HDACi induced invasion. **a** Active Rho pull down assay in A375 melanoma cells treated for 48 h with HDACi. Butyrate, TSA, Valproic acid and Vorinostat promoted active Rho downregulation. **b** Collagen invasion assay using Rho inhibitor C3T and ROCK inhibitor Y27. **c** HT −144 cells transfected with dominant negative for RhoA-T19N plasmid. Both inhibitors emulated the effect of HDACi on HT-144 invasion, as well as Rho downregulation by transfection. **d** Transfection efficiency measured as GFP transfected cells in cytometry. **e** GFP transfected cells as seen with fluorescence microscopy. *** Statistically significant difference from control, *p* < 0.001

In order to evaluate the possible role of small GTPases in the pro-invasive activity of HDACi, we performed a pull down assay for active RhoA in cell lysates from A375 cells untreated or treated for 48 h with HDACi. Our results show that there is a significant downregulation of active RhoA when cells are treated with butyrate, TSA and vorinostat (Fig. 7a), whereas a slight inhibition is observed with valproic acid.

Subsequently, we analyzed the role of the Rho/ROCK pathway in the invasive ability of melanoma cells. To this end, we performed a collagen invasion assay adding the Rho and ROCK inhibitors C3T and Y-27632, respectively. Both inhibitors emulate the pro-invasive effect of HDACi (Fig. 7 b). The collagen invasion assays performed using HT-144 cells transfected with dominant-negative RhoA (T19N) yielded similar results, confirming that the down-regulation of RhoA mimics the effects of HDACi in HT-144 cells.

## Discussion

Alterations in cell–cell adhesion, cytoskeleton reorganization and the acquisition of mesenchymal cell morphology are processes frequently observed during embryonic development, wound healing and several diseases, including cancer [31, 32]. These modifications may promote carcinoma cells to acquire invasion ability, which constitutes a critical step in tumor malignancy and metastasis [33, 34]. The E- to N-cadherin switch has been related to increased cell invasion, tumor progression and metastasis in melanoma and other carcinomas [30, 35–37].

Rho GTPase, which plays an important role in the regulation of the actin cytoskeleton, has also been associated with cancer invasion [38, 39]. Nevertheless, its role in tumor progression is still not clear, since the increased cell invasiveness has been associated with both the activation and the inhibition of Rho [30, 40–42].

The acetylation/deacetylation of chromosomal histones, regulated by HDACs and HATs, is an important epigenetic mechanism involved in the regulation of gene expression [43]. Currently, HDACi appear as promising antitumor drugs due to their ability to suppress proliferation and induce apoptosis [2, 44]. Nevertheless, there is some controversy about how HDACi affect the invasive potential of cancer cells. Some studies describe an anti-invasive activity of HDACi [2, 18–20, 44] whereas other authors affirm that these inhibitors increase invasion in several cancer cell lines [10, 21].

In the present study, we have demonstrated that several HDACi induce the up-regulation of N-cadherin and a significant inhibition of Rho activity in melanoma cell lines. Moreover, all these modifications lead to an increased invasiveness of melanoma cells into Matrigel and type I collagen matrices. Of the four HDACi examined, Butyrate, TSA and vorinostat are the most effective in inducing melanoma invasion; valproic acid show a pro-invasive activity on A375 but fail to induce invasion in HT-144. These results correlate with the modifications in the adhesion molecules and Rho activity observed in the HDACi treated cells. Butyrate, TSA and vorinostat significantly inhibit the activity of RhoA in melanoma cells, whereas valproic acid does not produce significant changes in this parameter. The distinct effects of Rho inhibition on cancer cell invasion has been related to the expression of its different isoforms [45] and the activity of the zinc-finger transcription factors Snai1 and Snail2 [41]. Moreover, it has been demonstrated that the down-regulation of Rho GTPase activity is able to induce collective cell migration through the stimulation of filopodia formation [42], in line with our morphological observations of HDACi treated melanoma cells. In this study we have also shown that the inhibition of the Rho/ROCK pathway by specific inhibitors or dominant-negative RhoA transfection leads to the same pro-invasive effect obtained with HDACi. This result suggests a direct implication of this pathway in the melanoma response to HDACi treatments.

Butyrate, TSA, valproic acid and vorinostat increase the association of N-cadherin with α-catenin, and thus, with the actin cytoskeleton. Butyrate does not alter the levels of E-cadherin linked to α-catenin, but does lead to the highest upregulation of functional N-cadherin. The effect of TSA and valproic acid on N-cadherin is lower than the effect of butyrate. However, they produce a significant downregulation of the E-cadherin association to alpha catenin. Surprisingly, vorinostat increases the association of both E-cadherin and N-cadherin to alpha catenin. Nevertheless, the dominance of the N-cadherin derived phenotype when the two cadherins are present has been previously demonstrated [46]. In the present study we demonstrate that the pro-invasive effect of butyrate, TSA and vorinostat on melanoma cells is completely abrogated through the inhibition of N-cadherin by a specific blocking antibody and siRNA. It is also noteworthy that several studies indicate that cadherin-mediated cell-cell adhesion and Rho small GTPase activity are reciprocally modulated [47, 48]. As we have previously mentioned, it is intriguing that the basal melanoma invasion observed in control cultures are not reduced by any of the methods used for N-cadherin inhibition. Our hypothesis is that this basal invasion is N-cadherin independent and that the increase in melanoma cell invasion in response to HDACi might be the result of some kind of signaling pathway triggered by the disturbance of the cadherin balance in favor to N-cadherin. Further research should be done in order to identify the mechanisms involved in this process. The identification of these mechanisms could help to understand the differences observed between *Matrigel* and collagen invasion assays, what could be related with the different composition of these ECM. Our results show that the proinvasive effect of HDACi is more effective on *Matrigel* than in collagen type I, what suggests that cell-matrix adhesion molecules with higher affinity for laminin or collagen type IV could be involved in the invasion process.

Taken all together, our results indicate that the heterogeneity of the melanoma cell response to the different HDACi, especially evident in HT-144 cells, is the result of a balance between the pro-apoptotic and pro-invasive effects of these drugs. We have found a reduction in the number of viable melanoma cells treated with HDACi which ranges from 3 to 25 %. This reduction is due to the combination of the well-known HDACi induced cell cycle arrest [17, 49] and the induction of apoptosis, which we have shown to be slightly increased after 24 h of exposure to the drugs. Accordingly, the failure of valproic acid to increase the number of invasive HT-144 cells in the collagen invasion assay could be partially explained by the significant reduction of viable HT-144 cell numbers produced by this drug, which also fails to inhibit the activity of RhoA. On the other hand, vorinostat had a antiproliferative effect which was similar to that of valproic acid on HT-144 cells but, at the same time, it produced the highest pro-invasive response in these cells. This apparently contradictory result could be explained by its very effective inhibition of the RhoA GTPase activity. Thus, the effect of HDACi on melanoma cell invasion is likely the result of a combination of several modifications in cell homeostasis in addition to apopotisis, N-cadherin induction or RhoA inhibition. Further research would be useful to clarify this issue.

## Conclusions

HDACi are currently in use in clinical trials against several cancer types [50]. Our results have demonstrated that HDACi induce melanoma cell invasion in vitro. According to the present study, the use of HDACi in melanoma patients could facilitate metastasis through the up-regulation of N-cadherin and the inhibition of RhoA activity. This finding sheds light on our understanding of the role of HDACi in tumor progression, but it also warrants caution regarding the use of these agents in the treatment of melanoma. Further investigations into the effects of HDACi should be carried out to elucidate the molecular mechanisms underlying their pro-invasive effects on melanoma cells. These investigations may provide the necessary insight to neutralize the potentially adverse effects of HDACi.

## Abbreviations

EMT: Epithelial to mesenchymal transition
GFP: Green fluorescent protein
HAT: Histone acetyl transferase
HDAC: Histone deacetylase
HDACi: Histone deacetylase inhibitor
HRP: Horseradish peroxidase
TSA: Trichostatin A
